# Robust Thiazole-Linked Covalent Organic Frameworks for Water Sensing with High Selectivity and Sensitivity

**DOI:** 10.3390/molecules29071677

**Published:** 2024-04-08

**Authors:** Kewei Wang, Zhaoxia Wu, Na Ji, Tingxia Wang, Yongxin Gu, Zhixiang Zhao, Yong Guo, Xiaoyan Wang, Zhifang Jia, Bien Tan

**Affiliations:** 1 Department of Chemistry and Chemical Engineering, Shanxi Datong University, Datong 037009, China; wuzhaoxia333@163.com (Z.W.); jina16301@163.com (N.J.); 17835202217@163.com (T.W.); guyongxin515@126.com (Y.G.); 18734398478@163.com (Z.Z.); ybsy_guo@163.com (Y.G.);; 2 Key Laboratory of Material Chemistry for Energy Conversion and Storage, Ministry of Education, Hubei Key Laboratory of Material Chemistry and Service Failure, School of Chemistry and Chemical Engineering, Huazhong University of Science and Technology, Wuhan 430074, China; bien.tan@hust.edu.cn

**Keywords:** covalent organic frameworks, hydrochromic property, proton-induced chromic mechanism, water sensing, high stability, water detection

## Abstract

The rational design of covalent organic frameworks (COFs) with hydrochromic properties is of significant value because of the facile and rapid detection of water in diverse fields. In this report, we present a thiazole-linked COF (TZ-COF-6) sensor with a large surface area, ultrahigh stability, and excellent crystallinity. The sensor was synthesized through a simple three-component reaction involving amine, aldehyde, and sulfur. The thiazole and methoxy groups confer strong basicity to TZ-COF-6 at the nitrogen sites, making them easily protonated reversibly by water. Therefore, TZ-COF-6 displayed color change visible to the naked eye from yellow to red when protonated, along with a red shift in absorption in the ultraviolet-visible diffuse reflectance spectra (UV-vis DRS) when exposed to water. Importantly, the water-sensing process was not affected by polar organic solvents, demonstrating greater selectivity and sensitivity compared to other COF sensors. Therefore, TZ-COF-6 was used to detect trace amounts of water in organic solvents. In strong polar solvents, such as *N*,*N*-dimethyl formamide (DMF) and ethanol (EtOH), the limit of detection (LOD) for water was as low as 0.06% and 0.53%, respectively. Even after 8 months of storage and 15 cycles, TZ-COF-6 retained its original crystallinity and detection efficiency, displaying high stability and excellent cycle performance.

## 1. Introduction

Meticulous structural design allows the creation of materials with the desired properties [1]. Covalent organic frameworks (COFs) are porous, crystalline organic materials constructed from rigid building blocks through covalent bonds [2]. In contrast to other polymers, COFs have predesignable structures, controllable synthesis, and manageable functionalization [3]. The synthesis of customized functional COFs can be achieved through topological design and polycondensation reactions, by carefully selecting suitable building blocks with specific geometries [4,5]. Therefore, COFs as highly ordered materials offer the advantages of tunable topology and pore size, nearly limitless functionalization capabilities, large surface area, and enduring porosity [6]. These advantages enable the application of COFs in gas storage and separation [7], energy storage [8], sensors [9], catalysis [10], and other applications [11]. Structurally, especially for the widely used two-dimensional (2D) COFs, the ordered layered structure provides a continuous one-dimensional nanoscale channel that allows guest molecules to freely access or interact with the functional sites of the channel wall [12]. Such one-dimensional channels and designable functional sites make COFs attractive platforms for molecular adsorption and sensing [13,14].

Water sensing as a detection technology for trace water has garnered significant attention in organic synthesis, cosmetics, pharmaceutical manufacturing, food-related industries, and anti-counterfeiting fields [15]. Compared to traditional Fischer titration [16] and chromatography [17], hydrochromic materials for water sensing, which undergo color changes upon interaction with water molecules, offer the advantages of shorter analysis time, simpler operation steps, and the use of nontoxic chemicals [18,19]. For COFs, functional groups containing O, N, or S heteroatoms can be easily introduced through bottom-up synthesis [20] or post-synthesis modification [21]. Heteroatom-containing groups, as functional sites, readily interact with water through hydrogen bonds or other interactions, potentially causing structural changes in the molecular structure of the material and resulting in a change in color. Therefore, COFs are promising hydrochromic materials for water-sensing applications. For example, Yang et al. recently investigated a solvatochromic luminescent COF for trace water determination, in which the sensor exhibited a strong color change dependent on solvent polarity, with good sensitivity and reversibility [22]. In contrast to solvatochromism, which relies on the stabilization of a single chromophore due to polarity-induced effects, Jhulki and co-workers developed a water-induced tautomeric COF that undergoes a color change mechanism from diiminol to iminol/*cis*-ketoenamine with a rapid response time [23]. A minority of acidochromic COFs can also respond to water, but no visible color change is observed during the sensing process, probably because of the weak basicity of the functional sites, which prevents them from bonding fully with the water proton and forming covalent bonds, leading to electronic structural variation [1,24,25,26,27]. While a few COFs have been used to detect water [28], hydrochromic COFs are still limited, and some issues persist: (1) The majority of the reported water-sensing COFs are B-O linked COFs, acylhydrazone, or imine COFs with low stability against water, especially under acidic or basic conditions. This chemical instability is extremely likely to result in poor cycling performance. (2) Low selectivity with water due to interference from strongly polar solvents. (3) The indistinguishable color change to the naked eye during the water-sensing process limits its practical use. Therefore, we have developed highly stable hydrochromic COFs for water sensing, featuring high selectivity, noticeable color change, and rapid response time.

**Figure 1:**
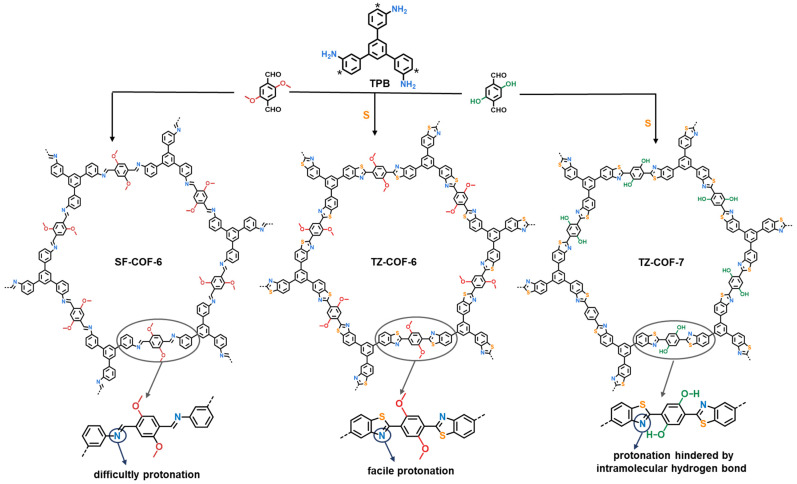
Synthesis of TZ-COFs and imine-linked COF (SF-COF-6). (TPB: 1,3,5-tris(3-aminophenyl)benzene. Electrophilic attack positions of sulfur in TPB are shown by star marks).

Thiazole-linked COFs (TZ-COFs) have been demonstrated to possess ultrahigh chemical stability against boiling water, organic solvents, strong acids/bases, and reductants [29,30,31,32,33]. Therefore, they can meet the demand in terms of stability for water sensing. In this study, we synthesized a methoxy-functionalized thiazole-linked COF (TZ-COF-6) via a simple multicomponent reaction involving 2,5-dimethoxy-terephthalaldehyde, amine, and sulfur (Figure 1). The resulting COF has a large surface area, excellent crystallinity, and high stability. The nitrogen-rich thiazole units endow COFs with basic sites that can be protonated by water, inducing structural changes which can be detected by spectroscopy and color changes which are visible to the naked eye. The electron-donating methoxy groups introduced by monomers not only protect TZ-COF-6 against tautomerization to yield *β*-ketoenamine-linked COFs during polymerization [23] but also increase the basicity of the N atoms in the thiazole units. As a consequence, TZ-COF-6 can respond to water by a proton-induced mechanism, accompanied by an instantaneous color change visible to the naked eye from yellow to red. The water-sensing process was nearly unaffected by other polar or protic solvents, displaying high selectivity and high sensitivity. Therefore, TZ-COF-6, a hydrochromic material, was used to detect trace amounts of water in different organic solvents. Even in strong polar solvents, such as DMF and EtOH, the LOD for water was as low as 0.06% and 0.53%, respectively. After eight months of storage and 15 cycles, TZ-COF-6 retained its original crystallinity and detection efficiency, displaying high stability and excellent cycle performance.

## 2. Results and Discussion

TZ-COFs were synthesized according to a previously described method [29], in which acetic acid as the catalyst and dimethyl sulfoxide as the oxidant were employed to construct TZ-COF via a one-pot multicomponent reaction of amine, aldehyde, and sulfur. However, only specific monomers of anthryl amine and naphthylamine, whose α-carbon located at the ortho-position of the amine group has a high electron density in order to easily electrophilic attack sulfur, were used in the reported method. In this study, we selected a moderately active amine (1,3,5-tris(3-aminophenyl)benzene, TPB) to react with 2,5-dimethoxy-terephthalaldehyde to prepare TZ-COF-6. TZ-COF-7 is a structural analog of TZ-COF-6 prepared from 2,5-dihydroxyterephthaldehyde. A sulfur-free imine COF (SF-COF-6) was prepared for structural and performance comparisons with TZ-COF-6.

### 2.1. Characterization of COFs

**Figure 2:**
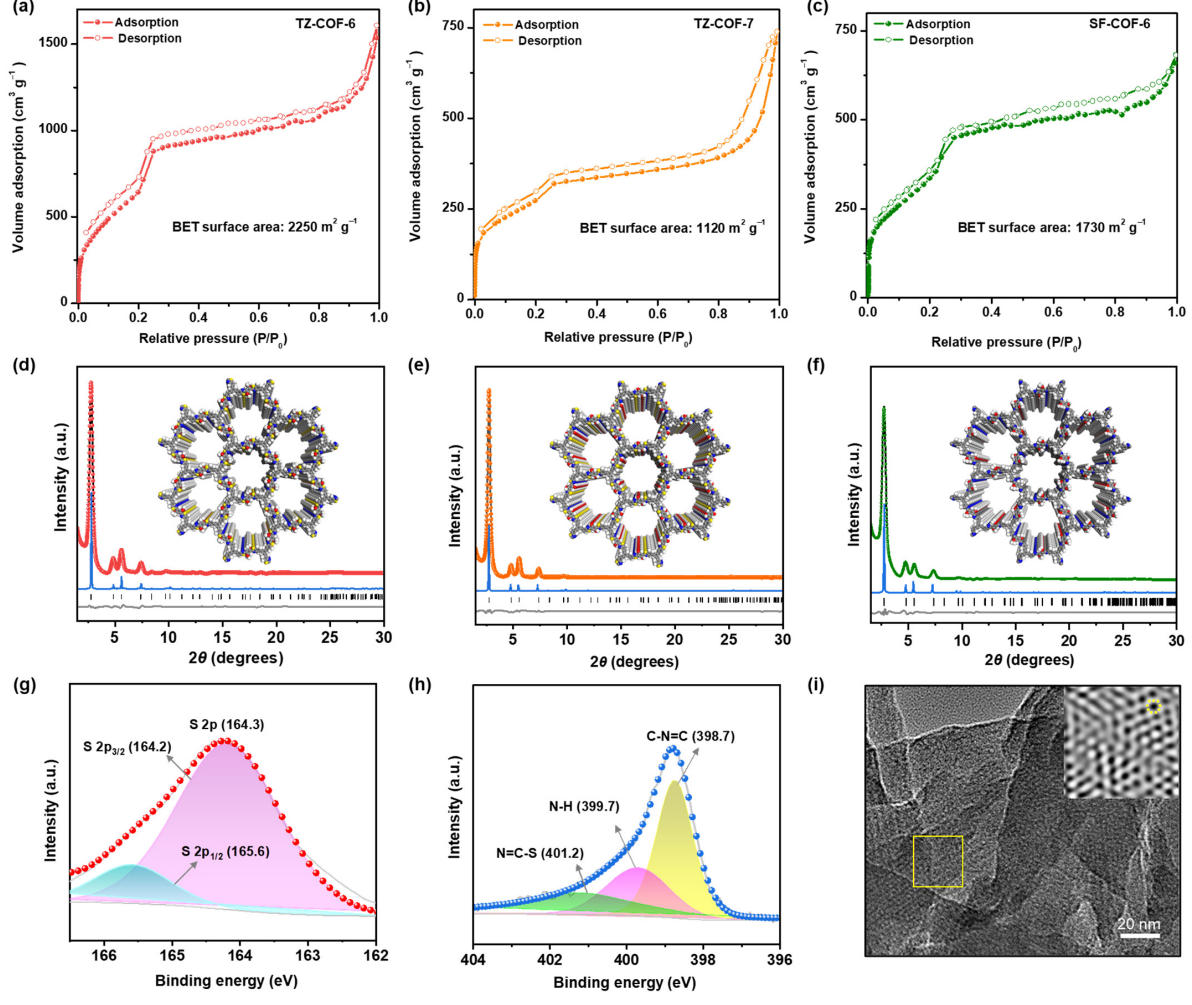
N_2_ adsorption−desorption isotherms of the three COFs (**a**–**c**). Experimental and simulated PXRD patterns for TZ-COF-6 (**d**), TZ-COF-7 (**e**), and SF-COF-6 (**f**) with perfectly eclipsed AA stacking patterns, shown parallel to the pore channel along the crystallographic *c* axis (inset). Experimental diffraction patterns (red, orange, or green), profiles calculated from Le Bail fitting (black) and residual (gray), and patterns simulated from the structural model (blue). Reflection positions are shown by tick marks. XPS curves of TZ-COF-6 in the region of S 2p (**g**) and N 1s (**h**). TEM image of TZ-COF-6 (**i**). The inset is a magnified image of the selected area (The yellow dotted circle indicates the hexagonal hole of TZ-COF-6).

The specific surface areas and porosities of the COFs were measured via nitrogen sorption analysis at 77.3 K. The Brunauer–Emmett–Teller (BET) surface area of TZ-COF-6 is 2250 m^2^ g^−1^, which is in fair agreement with the theoretical value of 2270 m^2^ g^−1^ (Figure 2a, Table S2). The BET surface areas of TZ-COF-7 and SF-COF-6 are 1120 and 1730 m^2^ g^−1^, respectively (Figure 2b,c). All COFs display type IV isotherms with slight hysteresis, indicating well-ordered mesopores. The pore size distribution calculated by density functional theory (DFT) shows a narrow pore size distribution for TZ-COF-6, TZ-COF-7, and SF-COF-6 of approximately 2.8, 2.7, and 3.2 nm, respectively (Figure S1), which are consistent with modeled eclipsed structures.

The powder X-ray diffraction (PXRD) patterns verified the structural regularity of the ordered layers within the three COFs (Figure 2d–f). The diffraction patterns are consistent with the eclipsed AA stacking arrangement rather than staggered AB stacking (Figure S2). Structural modeling showed that hexagonal channels were formed upon stacking the layers. The lattice parameters obtained after Le Bail refinements are in agreement with the structural models (Table S3) and the pore size distributions obtained from the gas adsorption analysis (Figure S1). For TZ-COF-6, refinement with the trigonal *P-3* space group (no. 147) reproduced the experimental XRD patterns with good agreement (R_wp_ = 1.52%, R_p_ = 4.14%). The clear diffraction peaks at approximately 2.8°, 4.8°, 5.6°, and 7.4° can be indexed to the (100), (110), (200), and (210) reflection planes (Figure 2d). The unit cell parameters were determined as *a* = *b* = 36.37 (3) Å, *c* = 3.54 (9) Å, *α* = *β* = 90.0°, and *γ* = 120°.

As shown in the Fourier-transform infrared (FT-IR) spectra, the weakening of the amine group (vibration absorptions at approximately 3435 and 3340 cm^−1^) and aldehyde group (at ~1682 cm^−1^) indicate the condensation occurrence of amino and aldehyde building blocks (Figure S3). The C=N characteristic peak of the thiazole linkage at 1593 cm^−1^ for TZ-COF-6 (1609 cm^−1^ for TZ-COF-7, Figure S4) can be observed, confirming the formation of thiazole rings [31,34]. The absorption peak at ~1620 cm^−1^ in SF-COF-6 is the typical imine-bond vibration (–C=N–) (Figure S5). Solid-state ^13^C nuclear magnetic resonance (NMR) provided further evidence of thiazole formation; the thiazole-carbon peak of S−C=N at ~167 ppm and the methoxy-carbon peak at 55 ppm appeared in TZ-COF-6 (Figure S6). The signals in the range 108–152 ppm are assigned to phenyl carbons. Elemental mapping of the TZ-COFs collected from the UV-vis energy dispersive spectrometer clearly shows the presence of C, N, O, and S elements with uniform distribution, revealing the successful occurrence of the multicomponent reaction (Figures S7 and S8). The construction of the thiazole ring was confirmed by X-ray photoelectron spectroscopy (XPS). For TZ-COF-6, the binding energies of S species at ~165.6 and ~164.2 eV observed in the S 2p spectra are attributed to S 2p_1/2_ and S 2p_3/2_ of the C–S moiety, respectively (Figure 2g) [29,32,33,34]. In the N 1s curves, two distinct bonding states, N of C=N–S (~401.2 eV) and C=N–C (~398.7 eV), point to the formation of thiazole rings (Figure 2h) [34]. The peak centered at ~399.7 eV is assigned to N–H of the residual amine groups. Similar binding energies of the S and N species were found in TZ-COF-7 (Figures S9 and S10). All the samples were subjected to scanning electron microscopy analysis to examine their morphologies (Figures S11–S13). Transmission electron microscopy (TEM) images demonstrate that the COFs have ordered pore structures (Figure 2i and Figures S14–S16). All the COFs possess high hermostability (T_dec_ > 350 °C), as measured by thermogravimetric analysis (Figure S17).

### 2.2. Hydrochromic Property for Water Sensing

**Figure 3:**
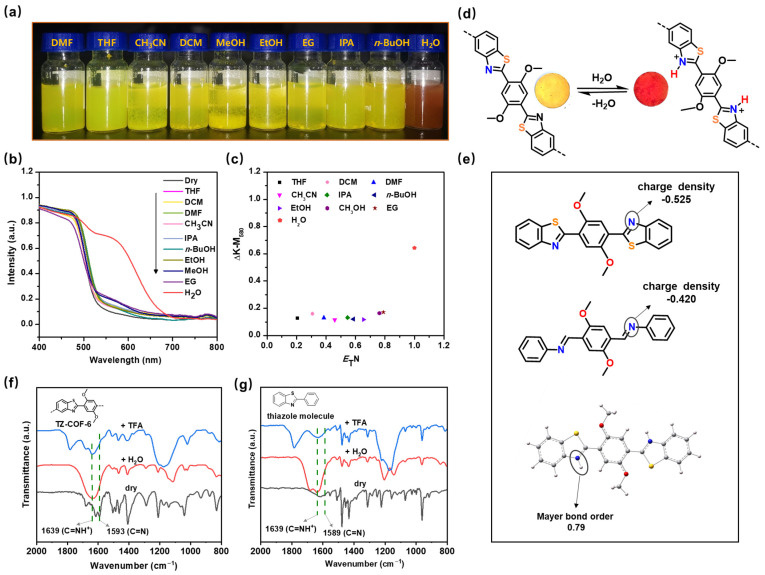
(**a**) Photograph of TZ-COF-6 soaked in different solvents with increasing polarity. (**b**) UV-vis DRS for TZ-COF-6 in different solvents. (**c**) A plot of change in the absorption strength (measured by the Kubelka−Munk function) at 580 nm (ΔK-M_580_) versus the *E*_T_N parameter. (**d**) Protonation of TZ-COF-6 with water causes a fully reversible color change from yellow to red. (**e**) Comparison of N charge density between the model compound of TZ-COF-6 and the model compound of SF-COF-6. The Mayer bond order between N and H in the model compound of TZ-COF-6 (yellow: S, blue: N, red: O, gray: C, and white: H). The charge densities and Mayer bond order were calculated by DFT using Gaussian 16. FT-IR curves of TZ-COF-6 (**f**) and thiazole molecule (**g**) treated with drying, water, and TFA, respectively.

The ultrahigh chemical stability and nitrogen-rich functional framework of TZ-COF-6 inspired us to explore its water response performance. The color of TZ-COF-6 changed from yellow to red when its dry form was watered (Figure 3a). The sensitivity of TZ-COF-6 to water is manifested by an instantaneous color change visible to the naked eye (Video S1). UV-vis DRS shows that dry TZ-COF-6 has an absorption onset of 528 nm, while it has a new band with an onset at approximately 670 nm when soaked in water (Figure 3b). To examine the selectivity for water and determine whether the color change was induced by the solvent effect [35], we soaked TZ-COF-6 in different solvents with increasing polarity. The sample retained its original yellow color in organic solvents (Figure 3a), although slight redshifts appeared in the absorption peaks of UV-vis DRS. Figure 3c shows the dependence of the absorption increment (measured by the Kubelka−Munk function) at 580 nm (ΔK-M_580_) on the solvent polarity parameter (*E*_T_N) [36,37]. ΔK-M_580_ has a nonlinear relationship with *E*_T_N in solvents with low-to-strong polarity: tetrahydrofuran (THF), dichloromethane (DCM), DMF, acetonitrile (CH_3_CN), isopropanol (IPA), *n*-butanol (*n*-BuOH), EtOH, methanol (MeOH), and ethylene glycol (EG). TZ-COF-6 displays quite a small absorption increment in these organic solvents, while there is a sharp increase in water. TZ-COF-6 is an excellent hydrochromic material with a high selectivity for water without interference from polar solvents. Additionally, the chromic phenomenon of TZ-COF-6 cannot be caused by isomerization, because the methoxy groups in the framework prevent TZ-COF-6 from undergoing iminol-to-ketoenamine tautomerism. Therefore, we deduced that the thiazole-N sites of TZ-COF-6 were responsible for its chromic response to water, given the lone pair of N atoms. To confirm this, the analog material TZ-COF-7, functionalized with a hydroxyl group, was prepared (Figure 1). As expected, no obvious color change of TZ-COF-7 was observed after the addition of water (Figure S18a), and only a slight red shift was shown in the UV-vis DRS (Figure S18b). The reason is that the thiazole N in TZ-COF-7 preferentially interacts with the hydroxyl through hydrogen bonding (O–H⋯N) using the lone pair of N atoms. Such intramolecular interactions hinder the interaction of COFs with water [38].

To investigate the effect of the thiazole structure on the chromic behavior, SF-COF-6, a structure equivalent to TZ-COF-6 but without S, was prepared for comparison. The UV-vis DRS of aqueous SF-COF-6 shows a minor red shift in contrast with dry COF, but no appreciable color change is observed visually (Figure S19), implying that thiazole units are important for hydrochromism. The influence could be that S atoms increase the basicity or electron density of the thiazole-N sites by the p-π conjugation effect in the thiazole ring, thereby easily holding water protons tightly. This inference was verified by DFT calculations for model compounds using Gaussian 16, in which the charge density of N in TZ-COF-6 (−0.525 eV) is denser than that of SF-COF-6 (−0.420 eV) (Figure 3e). The Mayer bond order between N (from thiazole) and H (from water) was calculated to be 0.79 (>0.1), revealing that the interaction between TZ-COF-6 and water is a covalent bond rather than hydrogen bonding (H–O–H⋯N). This result was directly verified by FT-IR measurement (Figure 3f). Compared to dry TZ-COF-6, the characteristic peak of C=N in the thiazole ring shifted from 1593 to 1640 cm^−1^ (C=NH^+^) [24] in the FT-IR spectra when aqueous TZ-COF-6 was tested. The same shift was detected when TZ-COF-6 was treated with a strong acid, trifluoroacetic acid (TFA). These results indicate that protonation of the C=N bonds caused a shift in the characteristic absorption peak to a high frequency. To further verify this, the thiazole molecule of 2-phenylbenzo-thiazole in dry, water-treated, and TFA-treated forms was also subjected to FT-IR measurements. It can be observed in Figure 3g that the appearance of vibration absorption peak of C=NH^+^ of thiazole at approximately 1639 cm^−1^ contrasts with the attenuation of the characteristic peak of C=N at 1589 cm^−1^ in the water-treated form (Figure S20). Moreover, the absorption edge shifts from 528 to 714 nm in the UV-vis DRS, with a color change from yellow to dark brown when dry TZ-COF-6 was treated with TFA (Figure S21a). The band gaps of TZ-COF-6, aqueous TZ-COF-6, and TFA-treated TZ-COF-6 are 2.35, 1.86, and 1.74 eV, respectively, calculated from UV-vis DRS by the linear-intercept method. The variation trend of the band gap is consistent with the red shift and color shift trends. Therefore, TZ-COF-6 exhibits a protonation-induced color shift and absorption red shift when exposed to water.

Based on the experimental and calculation results, we propose a mechanism of TZ-COF-6 for water sensing. As shown in Figure S22, for dry TZ-COF-6, the highest occupied molecular orbitals (HOMOs) and lowest unoccupied molecular orbitals (LUMOs) are delocalized over the entire framework. After protonation, the protonated thiazoles as new chromophores are stronger electron acceptors than their unprotonated counterparts. Thus, the energy of LUMO is lowered, and the LUMO is localized on the protonated thiazoles. The narrowing of the HOMO-LUMO band gap leads to an absorption red-shift in the UV-vis DRS and a color change (Figure 3d and Figure S21). The higher the protonation degree of the thiazoles, the narrower the HOMO-LUMO band gap and the darker the color displayed in TZ-COF-6. This inference is in agreement with the phenomenon that the color of TZ-COF-6 changes gradually from red (pH 7) to draw brown (pH 1) (Figure S21b).

The fast reversibility and instantaneous response process of materials for water sensing are important for their applications. As shown in Video S1, TZ-COF-6 immediately turns color from yellow to red when watered, and then quickly restores its original color after washing with a base (e.g., NaOH). This high-contrast color switching, visible to the naked eye for TZ-COF-6, is suitable for detecting trace water in organic synthesis and other relevant areas.

### 2.3. Detection of Trace Water in Organic Solvents

**Figure 4:**
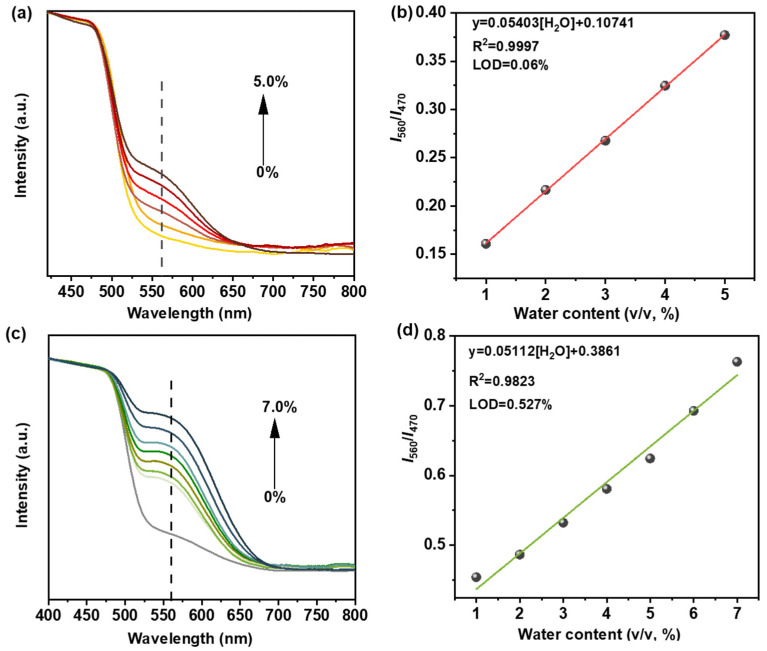
UV-vis DRS of TZ-COF-6 in DMF (**a**) and EtOH (**c**) with different water content, respectively. Calibration plots of absorption intensities ratio (at 560 nm/at 470 nm) of TZ-COF-6 in DMF (**b**) and EtOH (**d**) against water concentrations, respectively.

**Figure 5:**
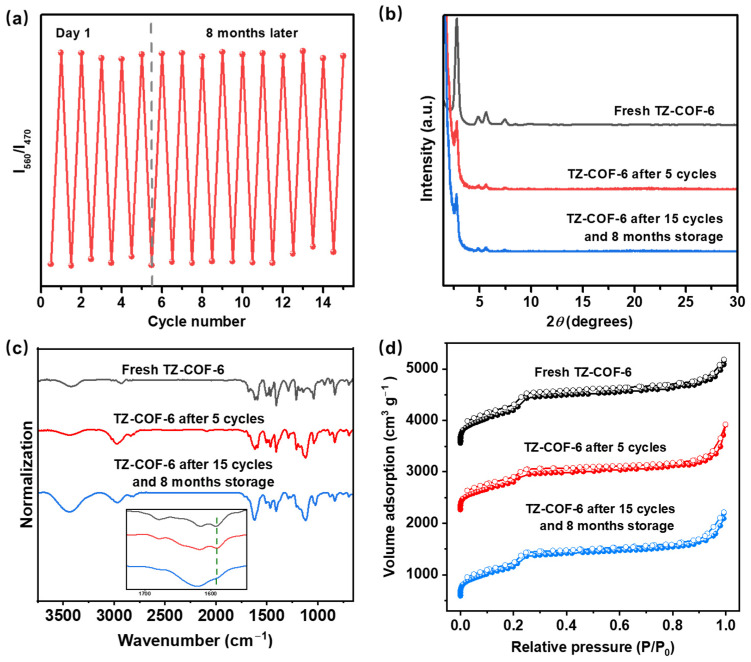
(**a**) Cycle test of TZ-COF-6 performed in DMF with a water content of 5.0%. The dashed line shows a gap of eight months of usage. The PXRD spectra (**b**), FT-IR (**c**), and N_2_ adsorption isotherms (**d**) of the fresh TZ-COF-6 and the recycled TZ-COF-6 after five cycles, and after eight months of ambient storage and 15 cycles, respectively.

Quantitative monitoring of trace water in organic solvents is of significant value in organic synthesis because water, as the most common impurity, always causes the failure of numerous chemical reactions requiring anhydrous harsh conditions. Because of the reversible high-contrast color switching towards water of stable TZ-COF-6 with high selectivity and fast response, we used it to detect trace water in organic solvents. Non-protonic solvents with different polarities (THF, DMF, CH_3_CN) and protonic solvents (CH_3_OH and EtOH) were selected for the study. New absorption peaks appeared at 560 nm in the presence of aqueous DMF in the UV-vis DRS of TZ-COF-6 (Figure 4a). The peaks gradually shifted to longer wavelengths and were significantly enhanced with the increase in water content from 0 to 5.0%. An excellent linear relationship between the absorption ratio and the amount of water in range of 0 to 5.0% is displayed with an R^2^ of 0.9997 (Figure 4b). The linear regression equation is *I*_560_/*I*_470_ = 0.0540[H_2_O] + 0.1074, and the LOD of trace water was calculated to be as low as 0.06% by 3*S*/*K* (S is the standard deviation for the DMF solution of TZ-COF-6 without water; K was obtained from the slope of the calibration curve). Notably, TZ-COF-6 showed a similar trend in the common protic solvent EtOH, in which the absorption peaks were red-shifted and significantly enhanced with the increase in water content from 0 to 7.0% (Figure 4c). The linear relationship equation and R^2^ are *I*_560_/*I*_470_ = 0.0511[H_2_O] + 0.3861 and 0.9823, respectively (Figure 4d). The LOD for water detection is 0.527%. Three other solvents, THF, CH_3_CN, and CH_3_OH, were also investigated, and the results are listed in Table S4, with LOD ranging from 0.340 to 0.735% (Figure S23). We conducted a cycle test in DMF with a water content of 5.0% to investigate the cycle life of the TZ-COF-6 sensor and found that it can be easily regenerated under alkaline conditions (e.g., NaOH). Even after more than eight months of ambient storage and 15 cycles (Figure 5a), no significant loss of sensing performance and crystallinity was observed (Figure 5b), and the structure was retained (Figure 5c,d), indicating high stability and good recyclability. The reported COFs as a sensing platform for monitoring water in organic solvents mostly depended on fluorescence signal detection, in which the fluorescent gradually quenched with the increase in water content, and the fluorescent intensity gradually decreased with the increase in the organic solvent polarity [22]. The weak fluorescent emission was hardly detected in strongly polar solvents, such as DMF or EtOH, thereby making it difficult to accurately monitor trace water. Compared to these fluorescence-imine COFs, TZ-COF-6 has the following advantages: (1) higher stability for better cycle life in polar solvents and (2) good selectivity for water; even in highly polar solvents (e.g., DMF and EtOH), due to lack of a significant positive solvatochromic effect, it is easy to accurately detect trace water. This can be illustrated by the comparison of the LOD and cycle numbers between TZ-COF-6 and other COFs (Table S5) [12,22,39,40,41].

## 3. Materials and Methods

Reagents and solvents were purchased from Heowns Biochem Technologies LLC (Tianjin, China) or Sinopharm Chemical Reagent Co., Ltd. (Shanghai, China). The monomer of TPB was purchased from Shanghai Kylpharm Co., Ltd. (Shanghai, China). Ultrapure water was obtained from the UPT-II-250L ultrapure water machine purchased from Sichuan Ulupure Ultrapure Technology Co., Ltd. (Chengdu, China). Instrumentation and materials and empirical parameters of solvent polarity (Table S1) are provided in Supplementary Materials.

### 3.1. Synthesis of TZ-COF-6, TZ-COF-7, and SF-COF-6

TPB (17.57 mg, 0.05 mmol), 2,5-dimethoxyterephthalaldehyde (14.56 mg, 0.075 mmol), and sulfur (14.43 mg, 0.45 mmol) were weighed into a 10 mL glass tube. Acetic acid (6 M, 0.10 mL), dimethyl sulfoxide (0.05 mL), o-dichlorobenzene (0.45 mL), and dimethylacetamide (0.50 mL) were then added into the aforementioned mixture. The sealed tube was placed in an oven at 120 °C for 72 h. The obtained yellow solid was isolated by centrifugation and washed with acetone (5 mL × 3) and tetrahydrofuran (THF, 5 mL × 3). The resulting solid was then subjected to Soxhlet extraction with toluene and THF as the solvent for 48 h, respectively. The powder was collected and dried at 80 °C for 12 h to yield TZ-COF-6 as a bright-yellow powder (21.50 mg, 63.06%). Elemental analysis (wt%): calc: C 68.40, H 4.42, N 6.14, O 7.01, S 14.04; found: C 67.52, H 4.68, N 7.56, O 9.15, S 11.09. TZ-COF-7 and SF-COF-7 were also prepared by this procedure. The detailed synthetic procedures are provided in Supporting Information.

### 3.2. Water Sensing

The ground polymer of TZ-COF-6 (15 mg) was placed in a quartz slide with grooves. Superdry THF (water ≤ 20 ppm, 0.1 mL) was added into the polymer and then sealed. The mixture was subjected to UV-vis DRS investigation by measuring the absorption of materials using dry TZ-COF-6 as a reference. DCM, DMF, CH_3_CN, IPA, n-BuOH, EtOH, MeOH, or EG was selected as Superdry solvent, respectively.

### 3.3. Water Detection in Different Solvents

Different volume fraction of mixed solvents of ultrapure water and Superdry THF (v_H2O_/v_THF_ = 0.0%, 0.2%, 0.4%, 0.6%, 0.8%, 1%, 2%, 3%, 4%, 5%) were prepared, respectively. Ground polymer of TZ-COF-6 (15 mg) was placed in the quartz slide with grooves. Each mixed solvent (0.1 mL) was mixed with TZ-COF-6 and then conducted to UV-vis DRS measurement. Other samples were prepared by Superdry DCM, DMF, CH_3_CN, IPA, n-BuOH, EtOH, MeOH, and EG using this procedure, respectively.

### 3.4. Cycle Testing of TZ-COF-6

Mixed solvent of pure water and Superdry DMF (v_H2O_/v_DMF_ = 5.0%, 0.1 mL) was added into dry TZ-COF-6 (15 mg). The sample was subjected to UV-vis DRS investigation at 560 nm and 470 nm, respectively. After the measurements, the wet sample was immersed in NaOH-EtOH solution (5.0 g/L, 5.0 mL) for 30 min. The solid collected by the filtration was dried at 90 °C for 5 h in the vacuum oven. The collected material was then directly used for the next cycle.

## 4. Conclusions

A robust thiazole-linked COF (TZ-COF-6) with hydrochromic properties was synthesized via a multiple-component method using amine, aldehyde, and sulfur. The resultant TZ-COF-6 possessed large surface area, excellent crystallinity, and superhigh stability. The thiazole framework and electron-donating methoxy groups endow the 2D COF with strong basicity that comes from the lone pair of thiazole nitrogen atoms. Therefore, TZ-COF-6 underwent a brilliant yellow-to-red protonation, as well as a red shift in the UV-vis DRS. Contrast experiments and DFT calculations revealed that the reversible color change was induced by the protonation of water at the thiazole nitrogen sites. TZ-COF-6 exhibited high selectivity towards the water, almost without interference from other solvents. Using this hydrochromic property and high selectivity, TZ-COF-6 was applied to detect trace water in organic solvents, giving LOD as low as 0.06% in DMF and an excellent cycle life (eight months and 15 cycles). The synthesis of a robust hydrochromic material was achieved in this work by the functional design of thiazole-linked COFs, which opens up a new application for thiazole-linked COFs and provides a candidate for stimuli-responsive chromic materials.

## Data Availability

Data are contained within the article and Supplementary Materials.

## Supplementary Materials

The following supporting information can be downloaded at: https://www.mdpi.com/article/10.3390/molecules29071677/s1: Figure S1: Pore size distributions of TZ-COF-6 (a), TZ-COF-7 (b), and SF-COF-6 (c); Figure S2: PXRD patterns of TZ-COF-6 (a), TZ-COF-7 (b), and SF-COF-6 (c); Figure S3: FT-IR spectra of TZ-COF-6 and monomers; Figure S4: FT-IR spectra of TZ-COF-7 and monomers; Figure S5: FT-IR spectra of SF-COF-6 and monomers; Figure S6: Solid-state ^13^C NMR of TZ-COF-6 (red), TZ-COF-7 (blue), and SF-COF-6 (green); Figure S7: Element mappings of EDS for TZ-COF-6 with S, N, O, and C; Figure S8: Element mappings of EDS for TZ-COF-7 with S, N, O, and C; Figure S9: XPS curves of TZ-COF-7 in the region of S 2p; Figure S10: XPS curves of TZ-COF-7 in the region of N 1s; Figure S11 (a,b): SEM images of TZ-COF-6; Figure S12 (a,b): SEM images of TZ-COF-7; Figure S13 (a,b): SEM images of SF-COF-6; Figure S14:TEM image of TZ-COF-6; Figure S15: TEM image of TZ-COF-7; Figure S16: TEM image of SF-COF-6; Figure S17: TGA curves of COFs; Figure S18: TZ-COF-7 for water sensing; Figure S19: SF-COF-6 for water sensing; (c) Plot of change in the absorption strength (measured by the Kubelka−Munk function) at 580 nm (ΔK-M_580_) versus the *E*_T_N parameter; Figure S20: (a) Comparison of the deprotonated and protonated TZ-COF-6 for FT-IR spectra; (b) Comparison of the deprotonated and protonated thiazole molecule for FT-IR spectra; Figure S21: UV-vis DRS plots, photographs, and band gap for TZ-COF-6 samples treated with drying, water, TFA, respectively; Figure S22: HOMO and LUMO frontier molecular orbits for different protonation degrees: unprotonated (a), partly protonated (b), fully protonated forms (c) of TZ-COF-6 model compound, calculated by gaussian 16; Figure S23: UV-vis DRS of TZ-COF-6 in THF (a), CH_3_CN (b), and CH_3_OH (c) with different water content, respectively. Calibration plots of absorption intensities ratio (at 560 nm/at 470 nm) of TZCOF-6 in THF (d), CH_3_CN (e), and CH_3_OH (f) against water concentrations, respectively; Table S1: Empirical parameters of solvent polarity *E*_T_(30) and normalized *E*_T_N values; Table S2: Surface area, porosity, and optical gap of COFs; Table S3: Crystal structure data for COFs; Table S4: Linear equation and LOD of TZ-COF-6 in solvents with different water content; Table S5: Comparison of TZ-COF-6 with other COFs in LOD and cycle in DMF or EtOH; Video S1: TZ-COF-6 protonation and deprotonation.
