# Supplementary material for: Robust Thiazole-Linked Covalent Organic Frameworks for Water Sensing with High Selectivity and Sensitivity
Source: Molecules. 2024 Apr 8;29(7):1677. doi: 10.3390/molecules29071677 (PMC11013684; doi:10.3390/molecules29071677)
Supplement: Supplementary file 1 [file molecules-29-01677-s001.zip › Supporting Information for proof-WKW.pdf]

Supporting Information

Robust thiazole-linked covalent organic frameworks for water sensing with high selectivity and sensitivity

Kewei Wang[a,1,*], Zhaoxia Wu[a,1], Na Ji[a], Tingxia Wang[a], Yongxin Gu[a], Zhixiang Zhao[a], Yong Guo[a], Xiaoyan Wang[b,*], Zhifang Jia[a], Bien Tan[b]

[a] Department of Chemistry and Chemical Engineering, Shanxi Datong University, Datong, 037009, China. Emails: wangkewei@sxdtdx.edu.cn; jiazf03110088@sxdtdx.edu.cn.

[b] Key Laboratory of Material Chemistry for Energy Conversion and Storage, Ministry of Education, Hubei Key Laboratory of Material Chemistry and Service Failure, School of Chemistry and Chemical Engineering, Huazhong University of Science and Technology, Wuhan 430074, China. Email: xiaoyan_wang@hust.edu.cn.

[1] Kewei Wang and Zhaoxia Wu contributed equally to this work.

Table of contents

1. **Instrumentation and Materials**…………………………………………… S2
2. **Synthetic procedures** ……………………………………………………… S5
3. **Characterization**…………………………………………………………… S8
4. **Water sensing** ………………………………………………………………S15
5. **Detection of trace water in organic solvents**………………………………S18
6. **References** ………………………………………………………………… S19

1. Instrumentation and Materials

2,5-Dimethoxyterephthalaldehyde, 2,5-dihydroxyterephthalaldehyde, trifluoroacetic acid (TFA), acetic acid (AcOH), 1,2-dichlorobenzene, 1-butanol (*n*-BuOH), methanol (MeOH), ethanol (EtOH), *N,N*-dimethylacetamide (DMAC), *N,N*-dimethylformamide (DMF), acetonitrile (CH_3CN), dioxane, tetrahydrofuran (THF), mesitylene, acetonitrile (CH_3CN), isopropanol (IPA), ethylene glycol (EG), *o*-dichlorobenzene (*o*-DCB), and dichloromethane (DCM) were purchased from Heowns Biochem Technologies LLC. Tian Jin. Sulfur, dimethyl sulfoxide (DMSO), and benzaldehyde were purchased from Sinopharm Chemical Reagent Co. Ltd. The monomer of 1,3,5-tris(3-aminophenyl)benzene was purchased from Shanghai Kylpharm Co. Ltd. Unless otherwise noted, reagents were obtained from commercial suppliers and used without further purification. Ultrapure water was obtained from the UPT-II-250L ultrapure water machine.

Fourier-transform infrared spectroscopy (FT-IR). FT-IR spectra were recorded on a FT-IR Bruker (VERTEX 70) spectrometer in the wavenumber range of 4000–400 cm^{-1} with KBr pellets.

Thermogravimetric analysis (TGA). TGA analysis was performed in a PerkinElmer Instruments Pyris1 TGA thermobalance under a flow of nitrogen by heating from room temperature to 1000 °C at a rate of 10 °C min^{-1}.

Solid-state ^{13}C Nuclear Magnetic Resonance (NMR). solid state ^{13}C NMR spectra were recorded on a WB 400 MHz Bruker Avance II spectrometer with the contact time of 2 ms (ramp 100) and pulse delay of 3 s.

Gas sorption analysis. The surface areas and N_2 adsorption isotherms of samples (77.3 K) were obtained using a Micromeritics ASAP 2020 volumetric adsorption analyzer. Before analysis, the samples were degassed at 110 °C for 8 h under vacuum (10^{-5} bar).

Powder X-ray diffraction (PXRD). Laboratory powder X-ray diffraction (PXRD) data were collected on a Bruker D8 Advance diffractometer equipped with a Cu Kα source (0.1 mm or 0.2 mm divergence slit, static air scatter screen) and a LynxEye XE detector. Kβ radiation was attenuated with a 0.0125 mm Ni filter.

X-ray photoelectron spectroscopy (XPS). XPS spectra in the region of C 1s, O 1s, N 1s, S 2p were performed on an ESCALAB MK II spectrometer (VG, UK).

Scanning electron microscopy (SEM). SEM images were carried out on Hitachi S-4800 cold field emission scanning electron microscope. Samples were prepared by depositing the dry powders on

15 mm Hitachi M4 aluminum stubs using an adhesive high-purity carbon tab before coating with a 2 nm layer of gold using an Emitech K550X automated sputter coater. Imaging was conducted at a working voltage of 3 kV and a working distance of 8 mm using a combination of upper and lower secondary electron detectors.

Transmission electron microscopy (TEM). TEM images were obtained on a Tecnai G20 microscope (FEI Corporation, Hillsboro, OR, USA) operated at an accelerating voltage of 200 kV.

Density Functional Theory (DFT) Calculations. Ground-state geometry optimizations on the TZ-COFs and SF-COF model compounds were performed using hybrid density functional theory B3LYP and the standard 6-31G(d) basis set, which has been demonstrated to describe molecular geometric and electronic properties reliably. Meanwhile, the frontier molecular orbital population of them from the computed structures were carried out at the same level of B3LYP/6-31G(d). All DFT computations were carried out with the Gaussian 16 program.

Ultraviolet-visible diffuse reflectance spectroscopy (UV-vis DRS). UV-vis DRS of the polymers were measured on a Shimadzu UV-2550 UV-vis spectrometer by measuring the absorption of powders in the solid state. The Kubelka-Munk equation can be written as

$$F(R_\infty) = \frac{K}{S} = \frac{(1-R_\infty)^2}{2R_\infty} \qquad \text{(S1)}$$

where K is the molar absorption coefficient, S is the scattering coefficient, R_∞ is the diffuse reflectance of an infinitely thick sample, $F(R_\infty)$ represents the Kubelka-Munk function.

Empirical parameters of solvent polarity from UV/Vis spectroscopic measurements. [1] Solvent polarity empirical parameter $E_T(30)$ values are based on the negatively solvatochromic pyridinium *N*-phenolate betaine dye as probe molecule, and the equation is

$$E_T(30)(kcal\, mol^{-1}) = \frac{28591}{\lambda_{max}\,(nm)} \qquad \text{(S2)}$$

where λ_{max} is the maximum of the longest wavelength, intramolecular charge-transfer π-π* absorption band of dye.

Normalized E_TN values are calculated from the equation (1-3)

$$E_TN = \frac{E_T(solvent)-E_T(TMS)}{E_T(water)-E_T(TMS)} = \frac{E_T(solvent)-30.7}{32.4} \qquad \text{(S3)}$$

Table S1. Empirical parameters of solvent polarity $E_T(30)$ and normalized E_TN values

Entry[a]	Solvent	$E_T(30)$ (kcal mol^{-1})	E_TN
1	THF	37.4	0.207
2	DCM	40.7	0.309
3	DMF	43.2	0.386
4	CH_3CN	45.6	0.460
5	IPA	48.4	0.546
6	*n*-BuOH	49.7	0.586
7	EtOH	51.9	0.654
8	MeOH	55.4	0.762
9	EG	56.3	0.790
10	H_2O	63.1	1.000

[a]: Measured at 25 °C and 1 bar.

Water sensing. The grinded polymer of TZ-COF-6 (15 mg) was placed in the quartz slide with grooves. Superdry solvent (water ≤ 20 ppm; THF, DCM, DMF, CH_3CN, IPA, *n*-BuOH, EtOH, MeOH, and EG; 0.1 mL) was added into TZ-COF-6 respectively, and then sealed. Using dry sample as a reference, the UV-vis DRS of TZ-COF-6 with different solvent was investigated on a Shimadzu UV-2550 UV-vis spectrometer by measuring the absorption of materials.

Water detection in different solvents. The grinded polymer of TZ-COF-6 (15 mg) was placed in the quartz slide with grooves. Different volume fraction of mixed solvents of ultrapure water and Superdry THF (v_{H_2O}/v_{THF} = 0.0%, 0.2%, 0.4%, 0.6%, 0.8%, 1%, 2%, 3%, 4%, 5%) were prepared respectively. The test sample was prepared by each mixed solvent (0.1 mL), sealed by quartz slide, and then conducted to UV-vis DRS measurement. Other samples were prepared by different solvent of Superdry DCM, DMF, CH_3CN, IPA, *n*-BuOH, EtOH, MeOH, or EG respectively according to the abovementioned procedures.

Cycle testing of TZ-COF-6. The dry sample of TZ-COF-6 (15 mg) and mixed solvents of pure water and Superdry DMF (v_{H_2O}/v_{DMF} = 5%, 0.1 mL) were subjected to UV-vis DRS measurements. The absorption intensity of each sample was investigated at 560 nm and 470 nm, respectively. After the measurements, the wet sample was immersed in NaOH-EtOH solution (5.0 g/L, 5 mL) for 30 min. The solid collected by the filtration and dried at 90 °C for 5 h in the vacuum oven. The collected material was then directly used in the next cycle test.

2. Synthetic procedures

2.1 General procedure for covalent organic framework (COFs)

Synthesis of TZ-COF-6

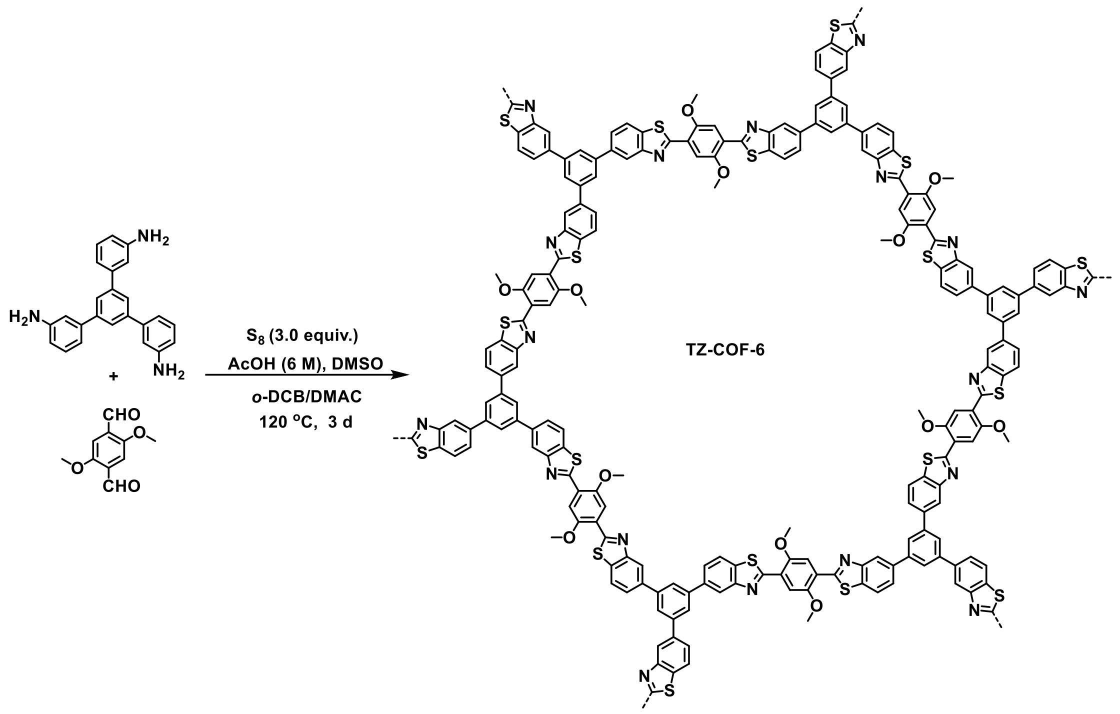

1,3,5-Tris(3-aminophenyl)benzene (17.57 mg, 0.05 mmol), 2,5-dimethoxyterephthalaldehyde (14.56 mg, 0.075 mmol), and sulfur (14.43 mg, 0.45 mmol) were weighed into a 10 mL glass tube (body length of 18 cm, neck length of 9 cm). AcOH (6 M, 0.10 mL), DMSO (0.05 mL), *o*-DCB (0.45 mL), and DMAC (0.50 mL) were then added into the mixture. The tube was flash frozen in a liquid nitrogen bath, evacuated to an internal pressure of 0.5 mbar and flame sealed, reducing the total length by ca. 10 cm. Upon warming to room temperature, the sealed tube was placed in an oven at 120 °C for 3 days, yielding a yellow solid. The tube was broken at the neck, and the yellow solid was isolated by centrifugation and washed with acetone (5 mL × 3) and THF (5 mL × 3). The resulting solid was dried, and then subjected to Soxhlet extraction with toluene and THF as the solvent for 48 h, respectively, to remove the trapped guest molecules. The powder was collected and dried at 80 °C for 12 h to yield TZ-COF-6 as a bright-yellow powder (21.50 mg, 63.06%). Elemental analysis (wt%): calc: C 68.40, H 4.42, N 6.14, O 7.01, S 14.04; found: C 67.52, H 4.68, N 7.56, O 9.15, S 11.09.

Synthesis of TZ-COF-7

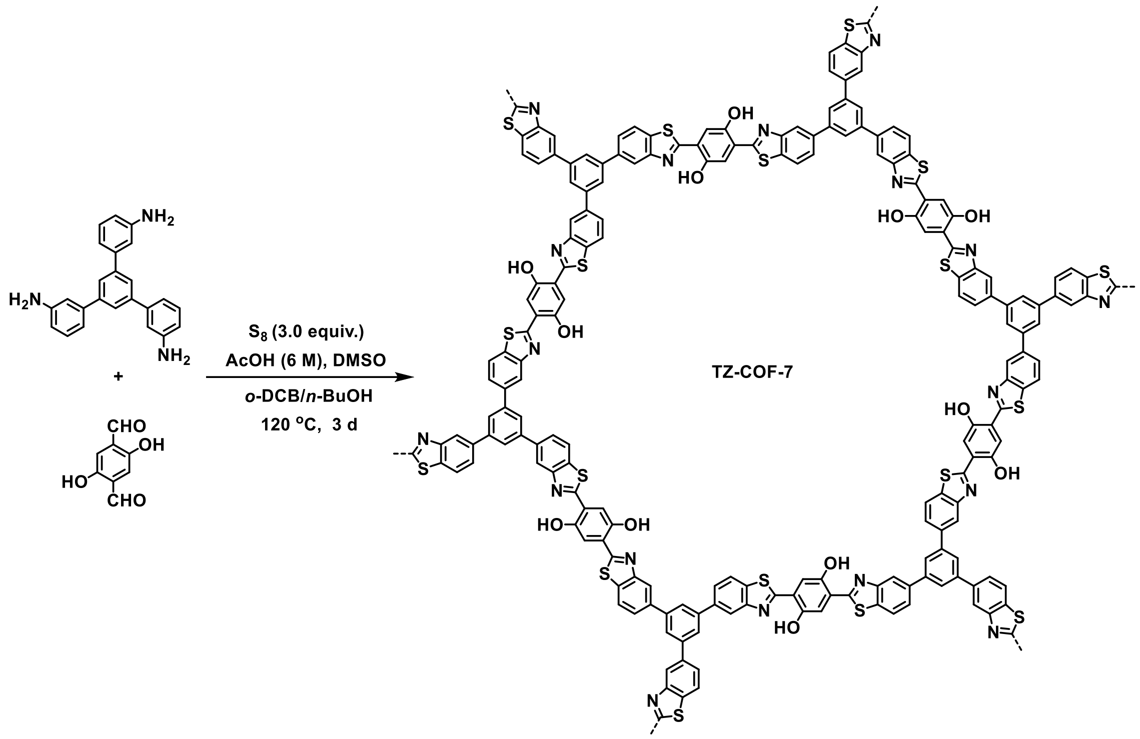

1,3,5-Tris(3-aminophenyl)benzene (17.57 mg, 0.05 mmol) and 2,5-dihydroxyterephthalaldehyde (12.45 mg, 0.075 mmol), and sulfur (14.43 mg, 0.45 mmol) were weighed into a 10 mL glass tube (body length of 18 cm, neck length of 9 cm). AcOH (6 M, 0.10 mL), DMSO (0.05 mL), *o*-DCB (0.45 mL), and *n*-BuOH (0.50 mL) were then added into the mixture. The tube was flash frozen in a liquid nitrogen bath, evacuated to an internal pressure of 0.5 mbar and flame sealed, reducing the total length by ca. 10 cm. Upon warming to room temperature, the sealed tube was placed in an oven at 120 °C for 3 days, yielding a red-brown solid. The tube was broken at the neck, and the red-brown solid was isolated by centrifugation and washed with acetone (5 mL × 3) and THF (5 mL × 3). The resulting solid was dried, and then subjected to Soxhlet extraction with toluene and THF as the solvent for 48 h, respectively, to remove the trapped guest molecules. The powder was collected and dried at 80 °C for 12 h to yield TZ-COF-7 as a red-brown powder (25.30 mg, 79.09%). Elemental analysis (wt%): calc: C 67.59, H 3.31, N 6.57, O 7.50, S 15.03; found: C 67.85, H 3.78, N 7.27, O 9.03, S 12.07.

Synthesis of SF-COF-6

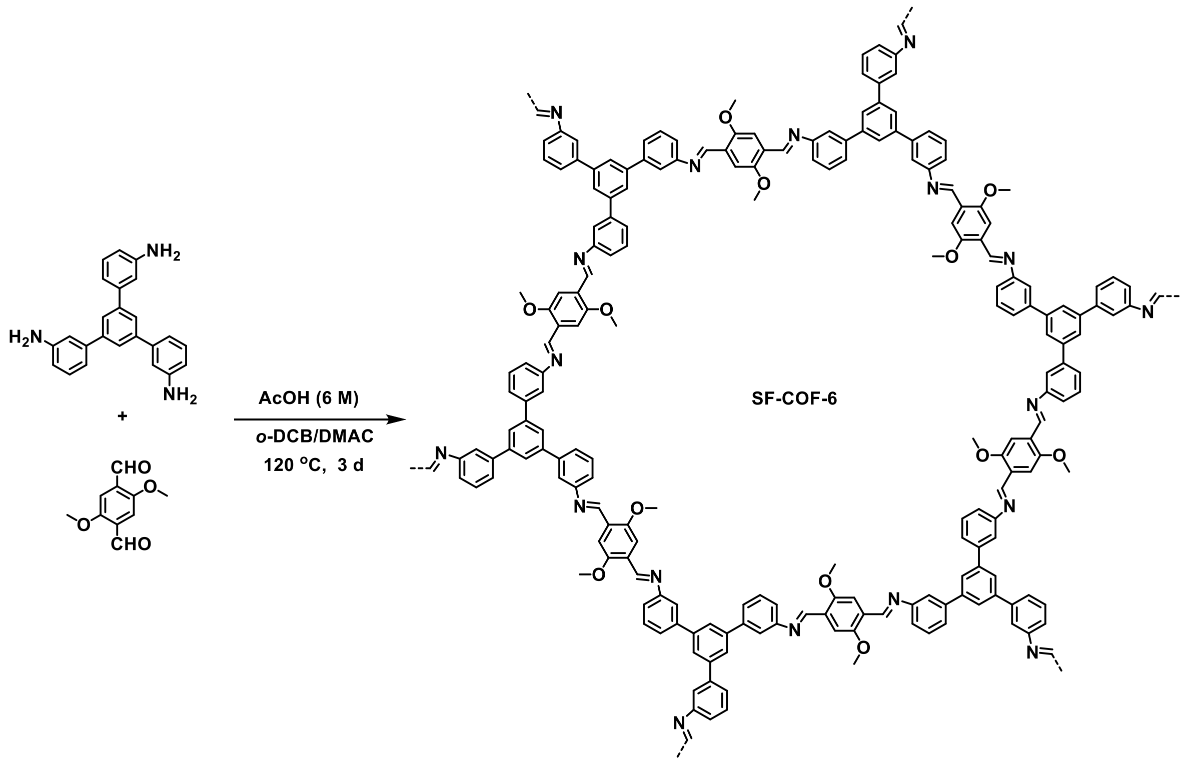

1,3,5-Tris(3-aminophenyl)benzene (17.57 mg, 0.05 mmol) and 2,5-dimethoxyterephthalaldehyde (14.56 mg, 0.075 mmol) were weighed into a 10 mL glass tube (body length of 18 cm, neck length of 9 cm). AcOH (6 M, 0.10 mL), *o*-DCB (0.50 mL) and DMAC (0.50 mL) were then added into the mixture. The tube was flash frozen in a liquid nitrogen bath, evacuated to an internal pressure of 0.5 mbar and flame sealed, reducing the total length by ca. 10 cm. Upon warming to room temperature, the sealed tube was placed in an oven at 120 °C for 3 days, yielding a bright-yellow solid. The tube was broken at the neck, and the bright-yellow solid was isolated by centrifugation and washed with acetone (5 mL × 3) and THF (5 mL × 3). The resulting solid was dried, and then subjected to Soxhlet extraction with toluene and THF as the solvent for 48 h, respectively, to remove the trapped guest molecules. The powder was collected and dried at 80 °C for 12 h to yield SF-COF-6 as a bright-yellow powder (18.80 mg, 63.87%). Elemental analysis (wt%): calc: C 79.17, H 5.62, N 7.10, O 8.11; found: C 78.88, H 5.86, N 6.87, O 8.38.

3. Characterization

3.1 Gas sorption

Table S2. Surface area, porosity, and optical gap of COFs

Sample	$S_{BET}{}^{a}$ (m^2 g^{-1})	Predicted $S_{BET}{}^{b}$ (m^2 g^{-1})	PV^{c} (cm^3 g^{-1})	Optical gap[d] (eV)
TZ-COF-6	2250	2270	2.48	2.35
TZ-COF-7	1120	1920	1.05	2.02
SF-COF-6	1730	2710	2.34	2.34

[a]: Surface area calculated from nitrogen adsorption isotherms at 77.3 K using BET equation. [b]: Surface area calculated from theoretical simulation by material studio. [c]: Total pore volume derived from adsorption isotherms at $P/P_0 = 0.095$, 77.3 K. [d]: Calculated by the linear intercept method from UV-vis DRS absorption spectrum.

3.2 Crystal structure data

Table S3. Crystal structure data for COFs

Sample	*a*, *b*, *c* (Å)	α, β, γ (°)	V (Å³)	Space group	R_{wp}, R_p (%)
TZ-COF-6	36.3729, 36.3729, 3.5487	90, 90, 120	4065.91	*P-3*	1.52, 4.14
TZ-COF-7	36.2855, 36.2855, 3.7545	90, 90, 120	4280.99	*P-3*	1.61, 3.59
SF-COF-6	36.6978, 37.6978, 3.7851	90, 90, 120	4414.58	*P-3*	4.85, 2.83

3.3 Pore size analysis of COFs

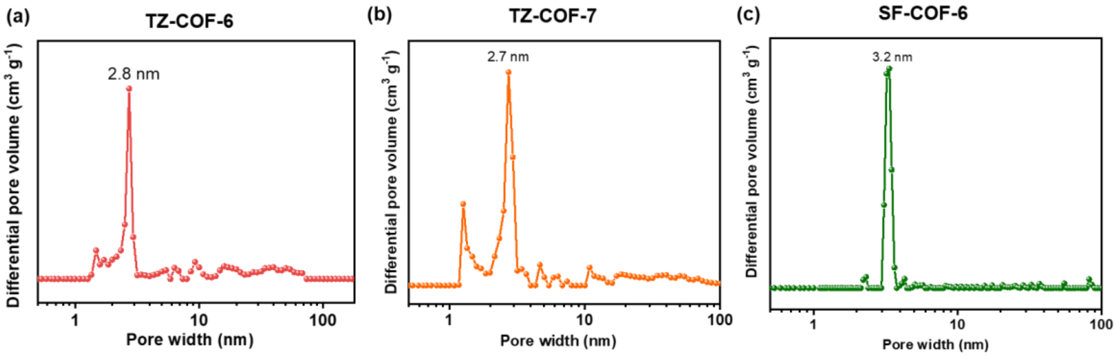

Fig. S1. Pore size distributions of TZ-COF-6 (a), TZ-COF-7 (b), and SF-COF-6 (c).

3.4 PXRD spectra of COFs

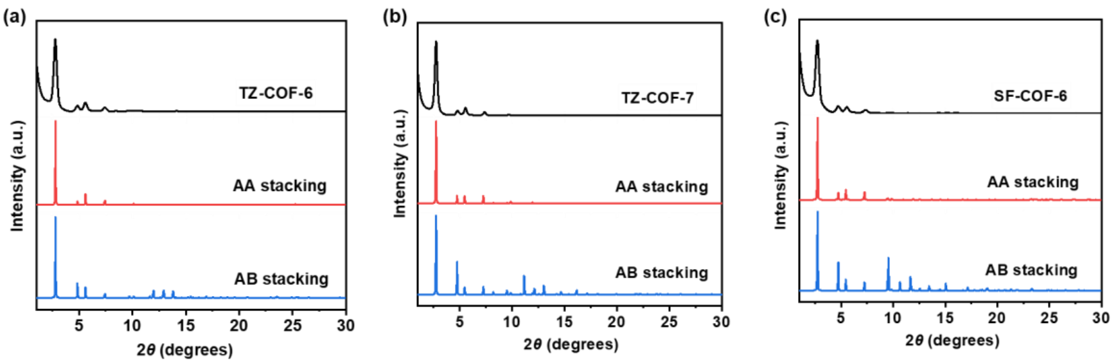

Fig. S2. PXRD patterns of TZ-COF-6 (a), TZ-COF-7 (b), and SF-COF-6 (c): experimental (black), calculated with the eclipsed (AA) stacking (red) and staggered (AB) stacking models (blue).

3.5 FT-IR spectra of COFs

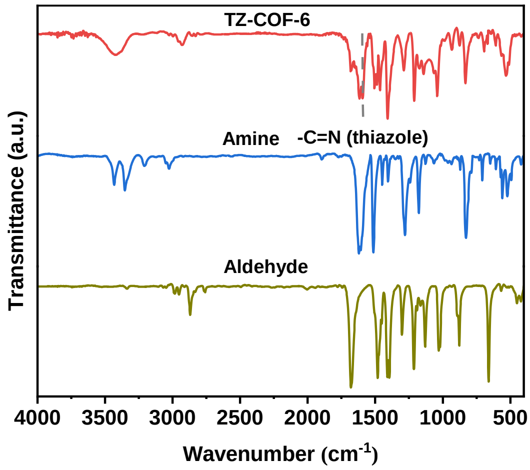

Fig. S3. FT-IR spectra of TZ-COF-6 and monomers.

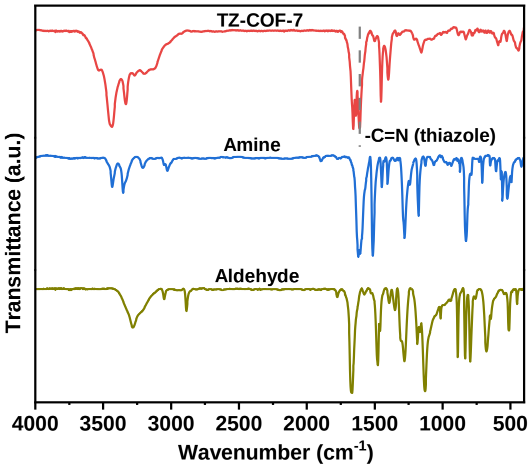

Fig. S4. FT-IR spectra of TZ-COF-7 and monomers.

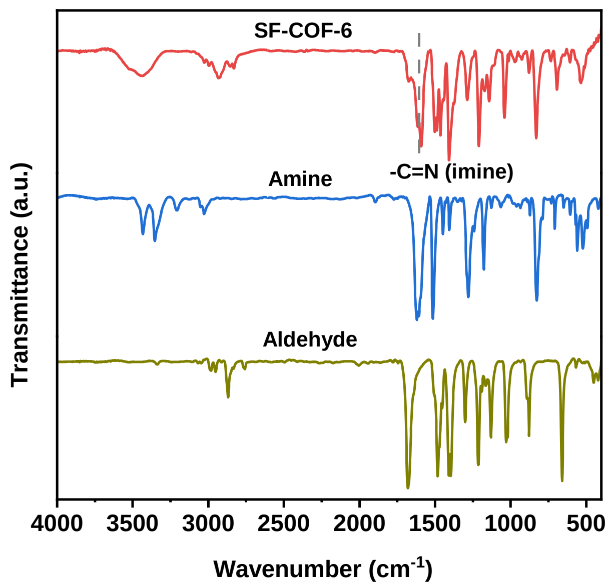

Fig. S5. FT-IR spectra of SF-COF-6 and monomers.

3.6 Solid-state ^{13}C NMR of COFs

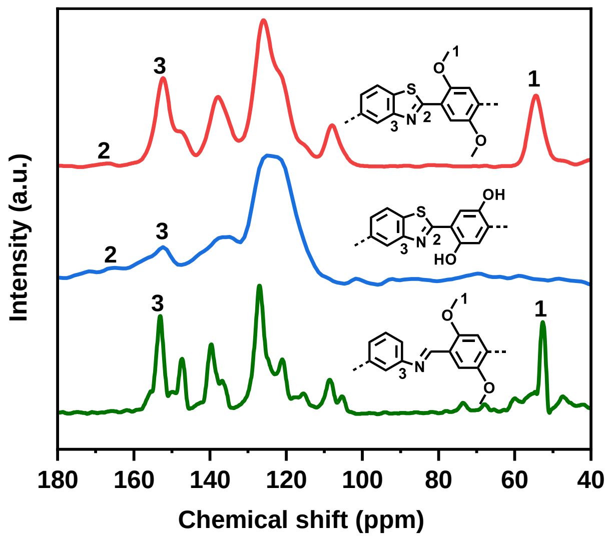

Fig. S6. Solid-state ^{13}C NMR of TZ-COF-6 (red), TZ-COF-7 (blue), and SF-COF-6 (green).

3.7 Element mappings of TZ-COFs

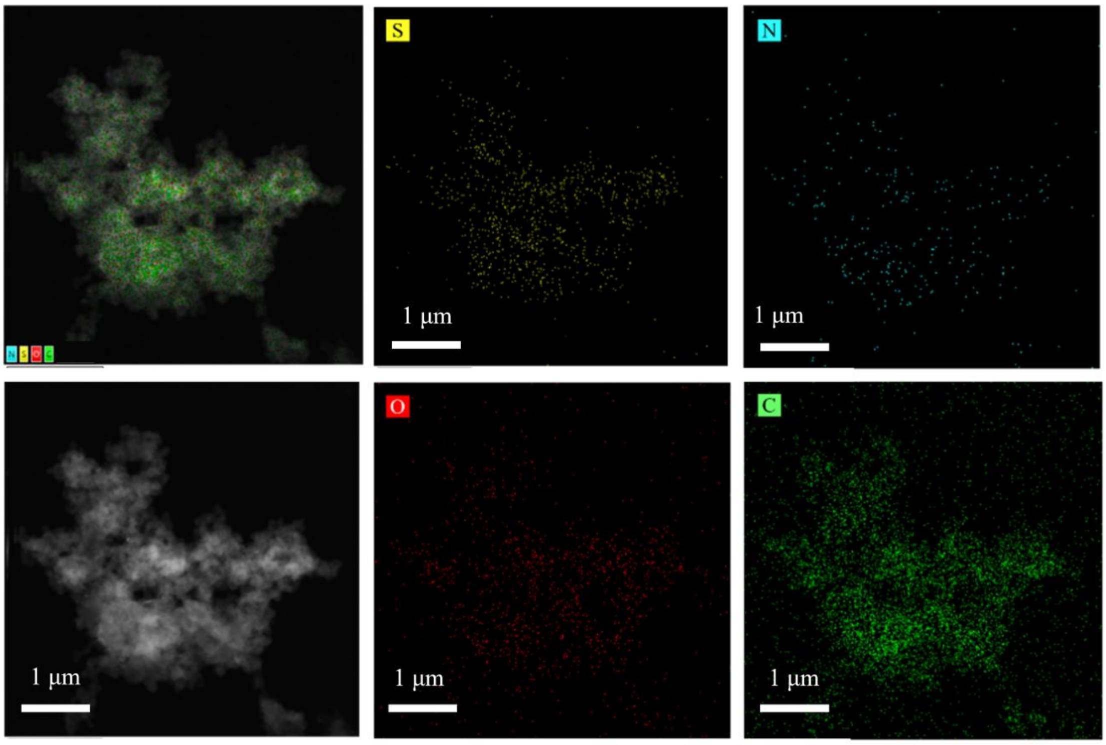

Fig. S7. Element mappings of EDS for TZ-COF-6 with S, N, O and C.

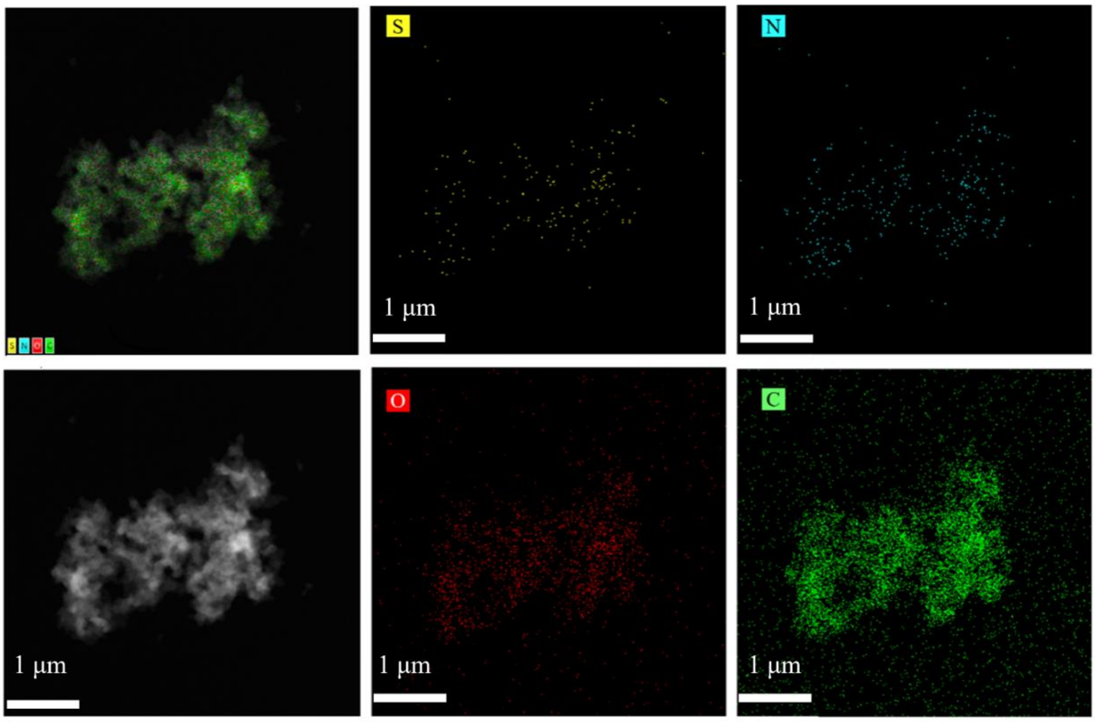

Fig. S8. Element mappings of EDS for TZ-COF-7 with S, N, O and C.

3.8 XPS pattens of COFs

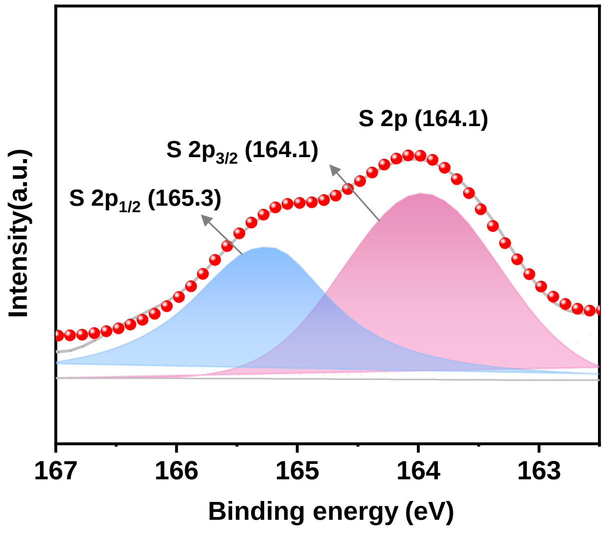

Fig. S9. XPS curves of TZ-COF-7 in the region of S 2p.

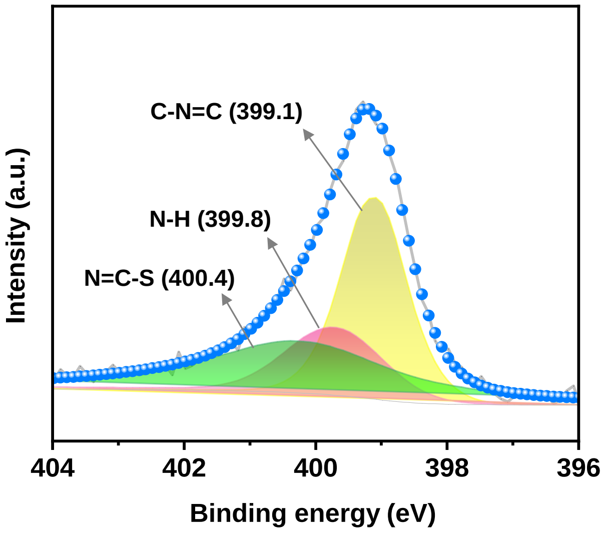

Fig. S10. XPS curves of TZ-COF-7 in the region of N 1s.

3.9 SEM of COFs

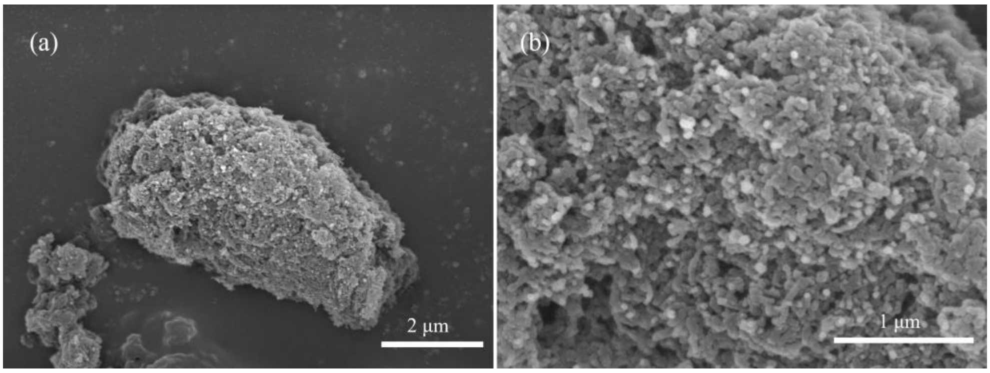

Fig. S11. SEM images of TZ-COF-6 with scale bars of 2 μm (a) and 1 μm (b).

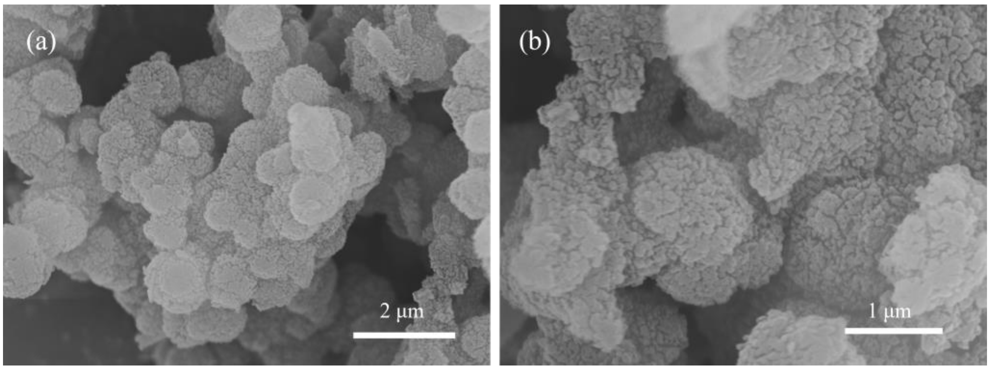

Fig. S12. SEM images of TZ-COF-7 with scale bars of 2 μm (a) and 1 μm (b).

.

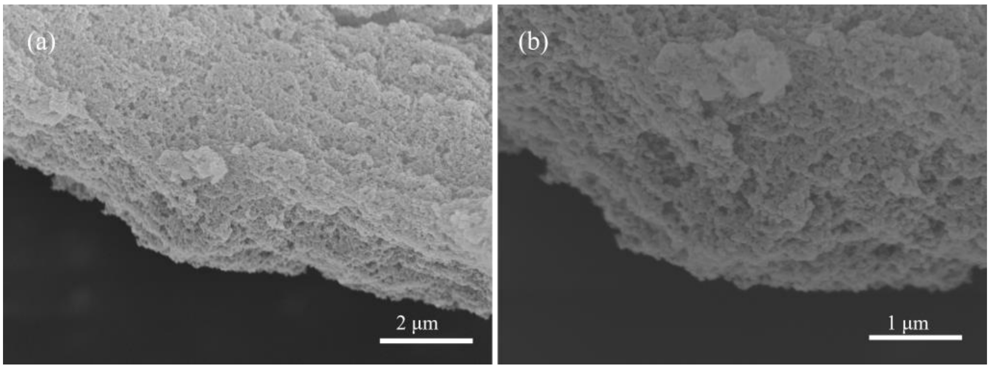

Fig. S13. SEM images of SF-COF-6 with scale bars of 2 μm (a) and 1 μm (b).

.

3.10 TEM of COFs

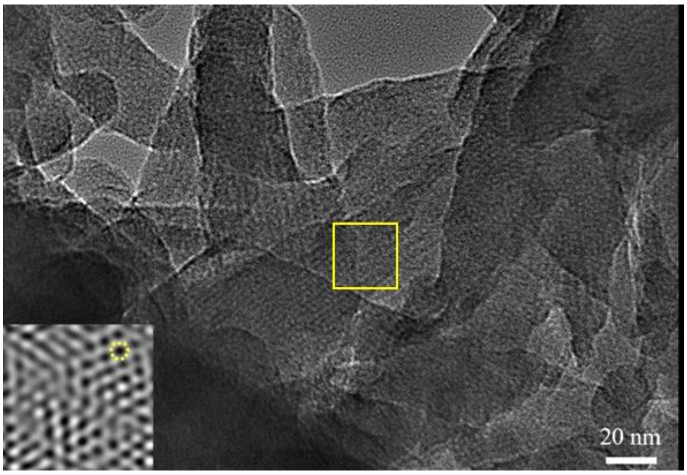

Fig. S14. TEM image of TZ-COF-6. The inset is magnified image of the selected area.

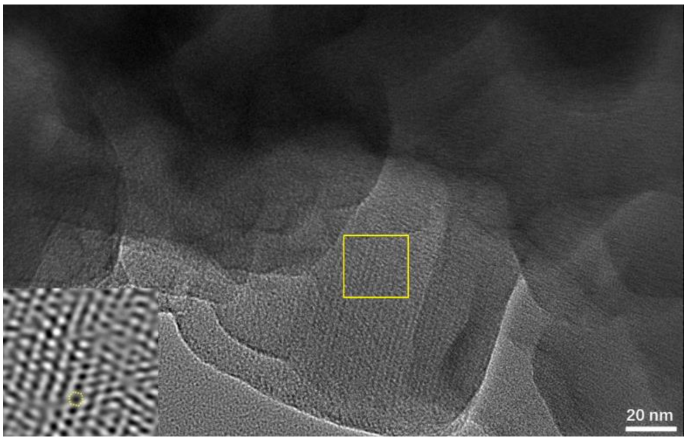

Fig. S15. TEM image of TZ-COF-7. The inset is magnified image of the selected area.

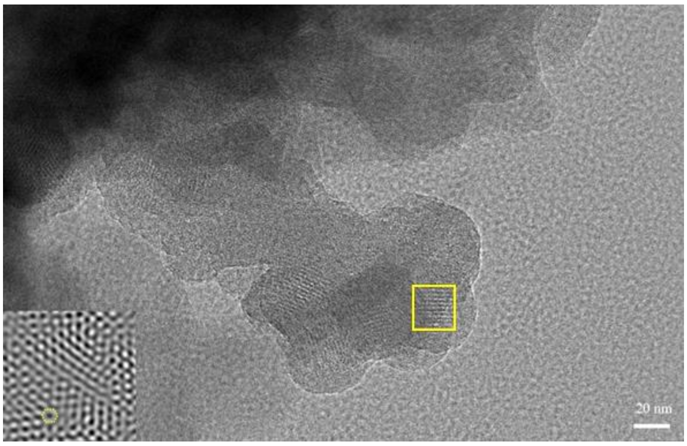

Fig. S16. TEM image of SF-COF-6. The inset is magnified image of the selected area.

3.11 TGA of COFs

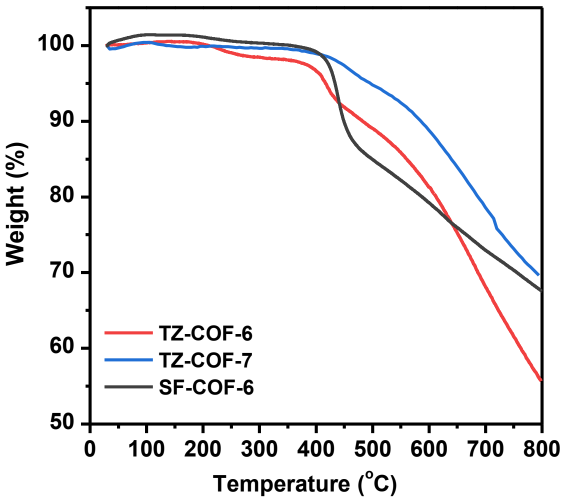

Fig. S17. TGA curves of COFs.

4. Water sensing

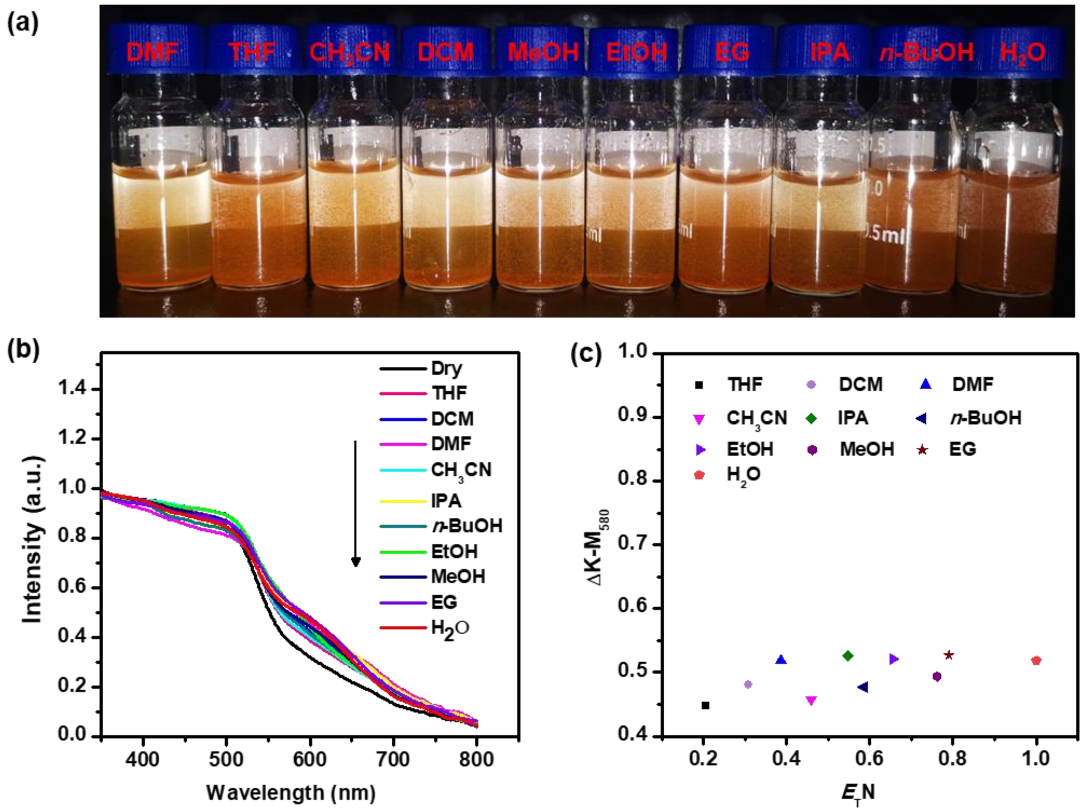

Fig. S18. TZ-COF-7 for water sensing. (a) Images of TZ-COF-7 in different solvents. (b) UV-vis DRS for TZ-COF-7 in different solvents (from weak to strong polarity). (c) Plot of change in the absorption strength (measured by the Kubelka−Munk function) at 580 nm (ΔK-M_{580}) versus the E_TN parameter.

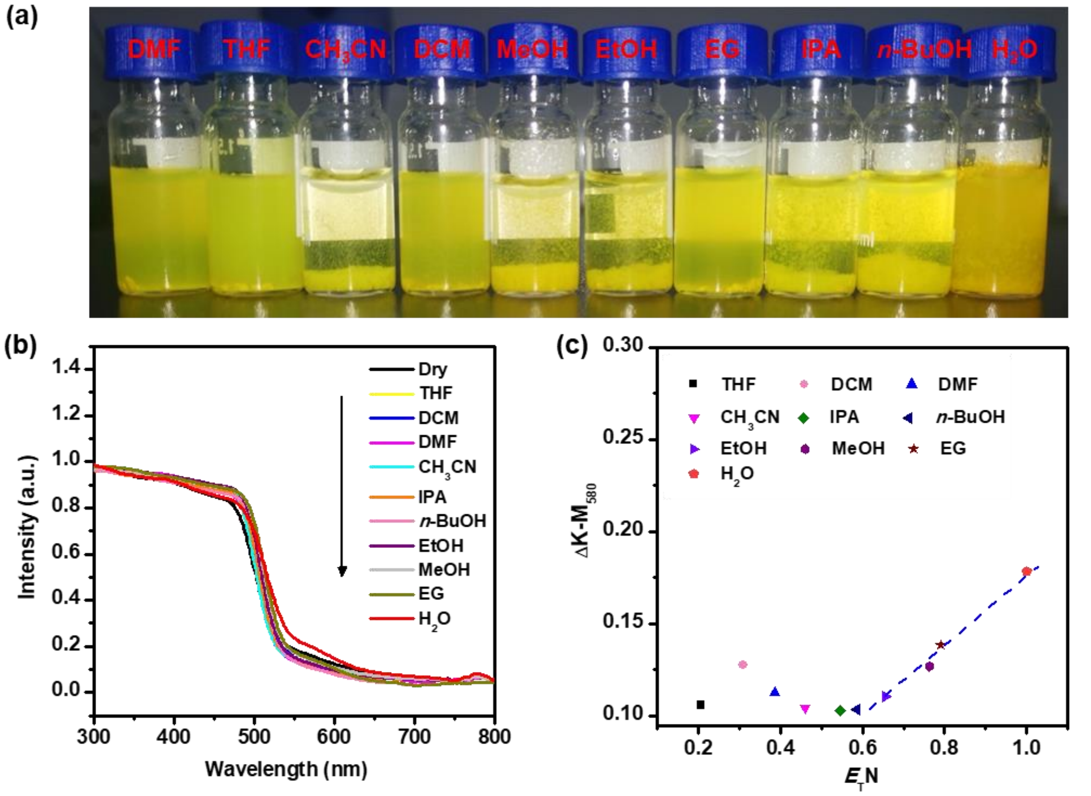

Fig. S19. SF-COF-6 for water sensing. (a) Images of SF-COF-6 in different solvents. (b) UV-vis DRS for SF-COF-6 in different solvents (from weak to strong polarity). (c) Plot of change in the absorption strength (measured by the Kubelka−Munk function) at 580 nm (ΔK-M$_{580}$) versus the E_TN parameter.

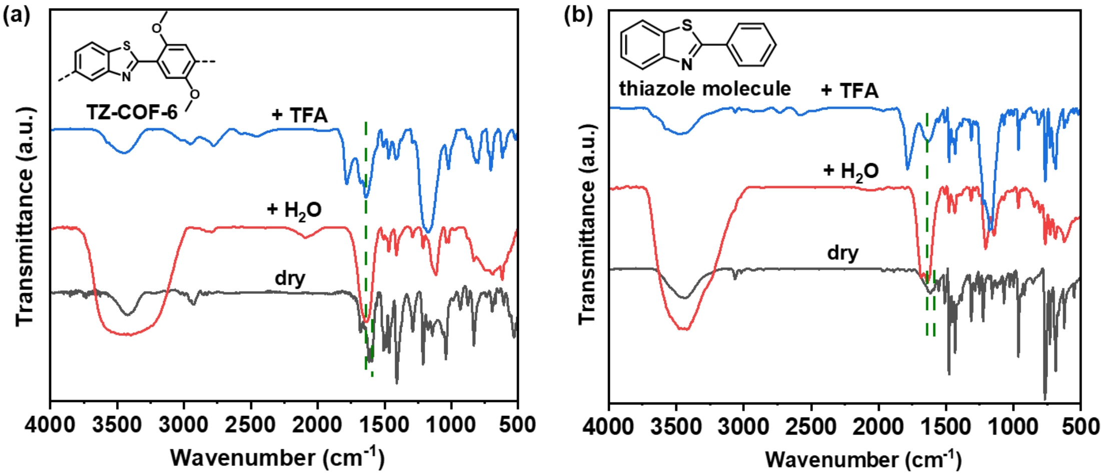

Fig. S20. (a) Comparison of the deprotonated and protonated TZ-COF-6 for FT-IR spectra. The unprotonated sample was treated with water and TFA respectively. The protonation leads to the appearance of the C=NH$^+$ stretching mode around 1640 cm^{-1}, accompanied by an attenuation of the thiazole C=N stretching mode around 1593 cm^{-1}. (b) Comparison of the deprotonated and protonated thiazole molecule for FT-IR spectra. The unprotonated molecule was treated with water and TFA respectively. The shift from (~1589 cm^{-1} of C=N) to (~1639 cm^{-1} of C=NH$^+$) is also observed in the model thiazole molecule.

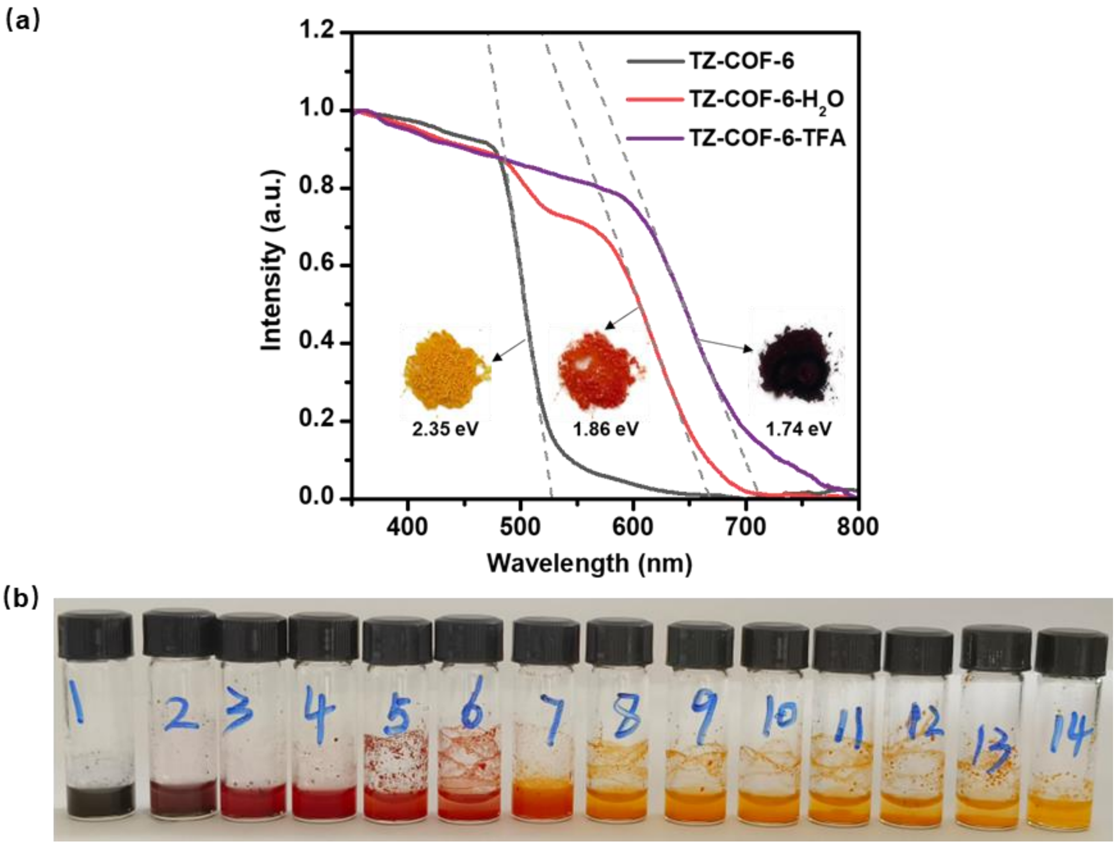

Fig. S21. (a) UV-vis DRS plots, photographs, and band gap for TZ-COF-6 samples treated with drying, water, TFA, respectively. (b) TZ-COF-6 color change in water solutions with different pH (from 1 to 14).

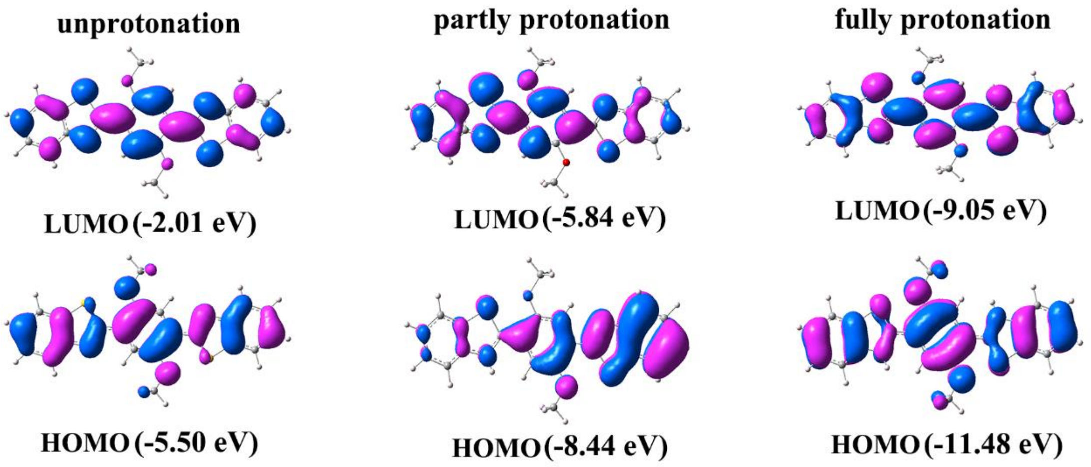

Fig. S22. HOMO and LUMO frontier molecular orbits for different protonation degrees: unprotonated, partly protonated, fully protonated forms of TZ-COF-6 model compound, calculated by gaussian 16.

5. Detection of trace water in organic solvents

Table S4. Linear equation and LOD of TZ-COF-6 in solvents with different water content

Solvent	Linear equation	R^2	Water content (v/v, %)	LOD (%)
THF	Y= 0.06266[H_2O] + 0.3157	0.9945	0.2–5	0.340
DMF	Y= 0.05403[H_2O] + 0.1074	0.9997	1–5	0.060
CH_3CN	Y= 0.09136[H_2O] + 0.2126	0.9921	0.2–5	0.408
CH_3OH	Y= 0.08523[H_2O] + 0.2996	0.9780	1–5	0.735
EtOH	Y= 0.05112[H_2O] + 0.3861	0.9823	1–7	0.527

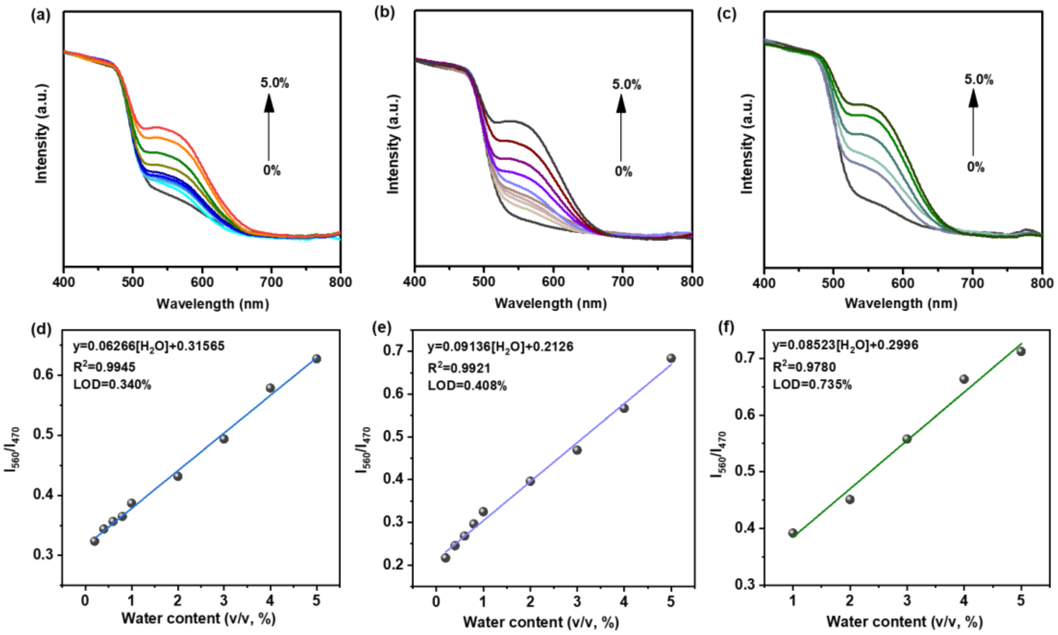

Fig. S23. UV-vis DRS of TZ-COF-6 in THF (a), CH_3CN (b), and CH_3OH (c) with different water content, respectively. Calibration plots of absorption intensities ratio (at 560 nm/at 470 nm) of TZ-COF-6 in THF (d), CH_3CN (e), and CH_3OH (f) against water concentrations, respectively.

Table S5. Comparison of TZ-COF-6 with other COFs in LOD and cycle in DMF or EtOH

Material	Type	Solvent	LOD (v/v, %)	Cycle	reference
TZ-COF-6	COF	DMF	0.06	15[a]	this work
		EtOH	0.527	N/A	
Pythz-COF	COF	DMF	2.94	5	[2]
Pyurea-COF	COF	DMF	1.7	N/A	
DpTTA-COF	COF	DMF	0.11	3	[3]
COFs	COF	DMF	0.262	N/A	[4]
		EtOH	0.424	N/A	
TzDa	COF	EtOH	0.034	9	[5]
Tb^{3+}@p-Ds/MOF	carbon dots	DMF	0.33	N/A	[6]

[a]: The sample was stored at ambient conditions for eight months after five cycles, then followed the next 9 cycles.

References

[1] Reichardt, C. Solvatochromic dyes as solvent polarity indicators. *Chem. Rev.* **1994**, *94*, 2319–2358.

[2] Jiang, S.; Meng, L.; Ma, W.; Pan, G.; Zhang, W.; Zou, Y.; Liu, L.; Xu, B.; Tian, W. Dual-functional two-dimensional covalent organic frameworks for water sensing and harvesting. *Mater. Chem. Front.* **2021**, *5,* 4193−4201.

[3] Yang, L.; Wang, Y.; Cui, E.; Yang, X.; Yuan, X.; Pu, J.; Zhao, Y.; Xie, H.; Hu, J.; Liu, J.; Liu, Y. Solvatochromic covalent organic frameworks for the low-level determination of trace water in organic solvents, *Sensor. Actuat. B-Chem.* **2023**, *378*, 133134.

[4] Chen, Y.; Zhang, C.; Xie, J.; Li, H.; Dai, W.; Deng, Q.; Wang, S. Covalent organic frameworks as a sensing platform for water in organic solvent over a broad concentration range. *Anal. Chim. Acta* **2020**, *1109*, 114–121.

[5] Qian, H.; Dai, C.; Yang, C.; Yan, X. High-Crystallinity covalent organic framework with dual fluorescence emissions and its ratiometric sensing application. *ACS Appl. Mater. Interfaces* **2017**, *9*, 24999–25005.

[6] Wu, J.; Yan, B. A dual-emission probe to detect moisture and water in organic solvents based on green-Tb^{3+} post-coordinated metal-organic frameworks with red carbon dots. *Dalton Trans.* **2017**, *46*, 7098–7105.
